# Supplementary material for: Functional Characterization of the GNAT Family Histone Acetyltransferase Elp3 and GcnE in Aspergillus fumigatus
Source: Int J Mol Sci. 2023 Jan 22;24(3):2179. doi: 10.3390/ijms24032179 (PMC9916960; doi:10.3390/ijms24032179)
Supplement: Supplementary file 1 [file ijms-24-02179-s001.zip › Figure S1.pdf]

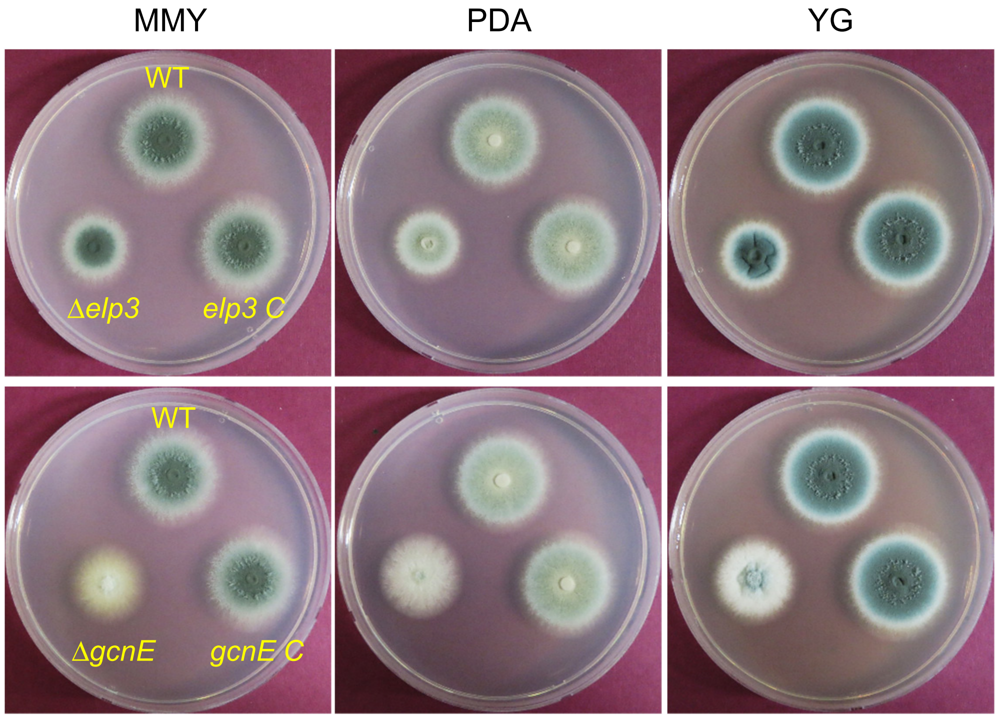

Figure S1. Colony photographs of WT, Δ*elp3*, Δ*gcnE* and relevant complemented strains. Each strain was point-inoculated on various solid media and grown for 2 days.
