# Supplementary material for: Functional Characterization of the GNAT Family Histone Acetyltransferase Elp3 and GcnE in Aspergillus fumigatus
Source: Int J Mol Sci. 2023 Jan 22;24(3):2179. doi: 10.3390/ijms24032179 (PMC9916960; doi:10.3390/ijms24032179)
Supplement: Supplementary file 1 [file ijms-24-02179-s001.zip › Figure S2.pdf]

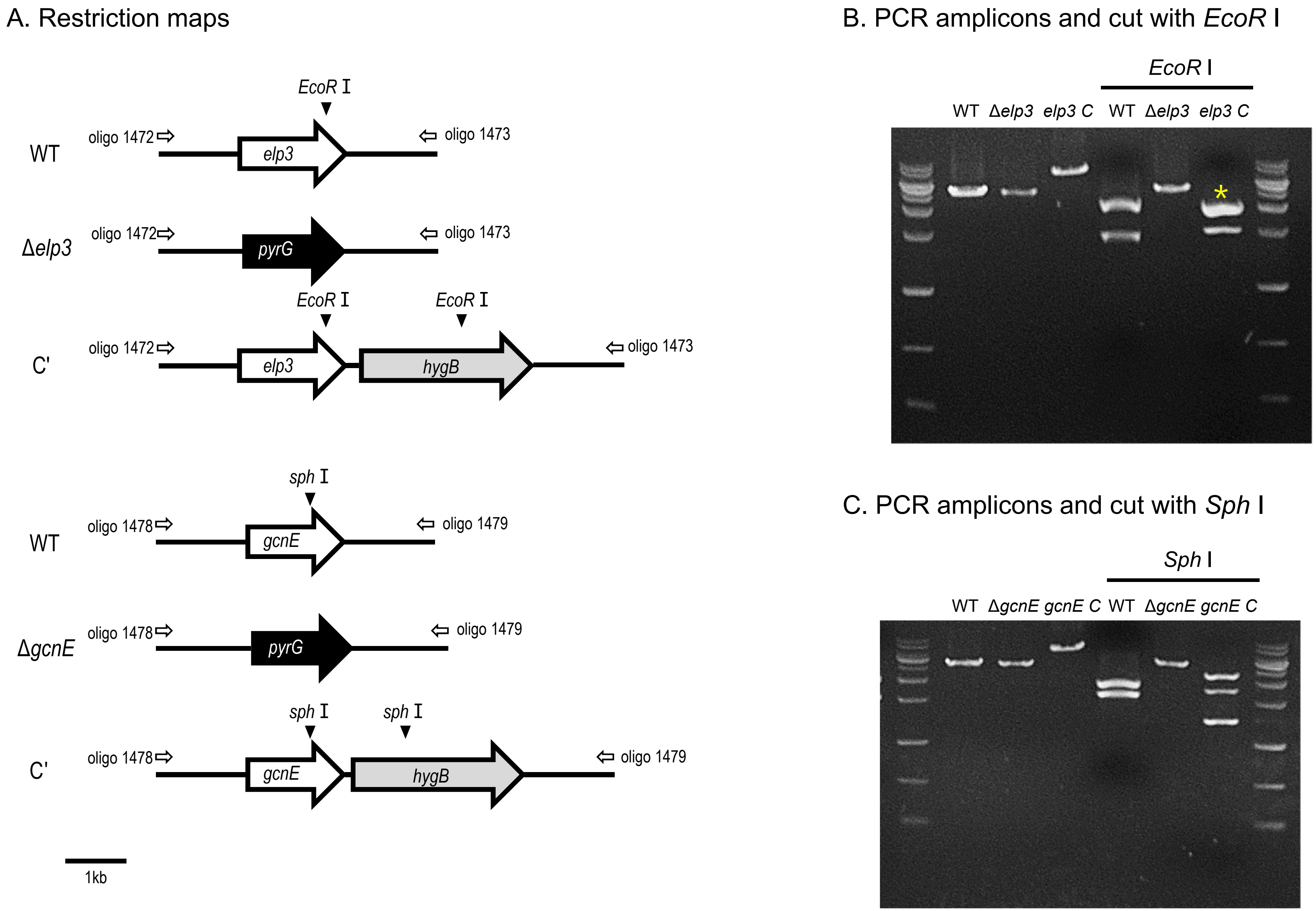

Figure S2. Confirmation of WT, mutants, and complemented strains. (A) Schematic illustration for *elp3* and *gcnE* genes region of WT, mutants, and complemeted strains, respectively. (B) PCR results for deletion and complementation of *elp3* and *EcoR* I digestion pattern of the individual amplicons. (C) PCR results for deletion and complementation of *gcnE* and *Sph I* digestion pattern of the individual amplicons. * indicates two DNA fragments (2,761 and 2,666 bp).
