# Supplementary material for: Functional Characterization of the GNAT Family Histone Acetyltransferase Elp3 and GcnE in Aspergillus fumigatus
Source: Int J Mol Sci. 2023 Jan 22;24(3):2179. doi: 10.3390/ijms24032179 (PMC9916960; doi:10.3390/ijms24032179)
Supplement: Supplementary file 1 [file ijms-24-02179-s001.zip › Table S1.pdf]

Table S1. Oligonucleotides used in this study.

Name	Sequence (5'→3')[a]	Purpose
oligo 256	TTCCAAGCAGAGCTTGTCAC	5' *brlA* for qRT-PCR
oligo 257	CCAGGTTCTTTGCACTTGAA	3' *brlA* for qRT-PCR
oligo 271	AAATCCATCACATCCACCCT	5' *gliZ* for qRT-PCR
oligo 272	GGTTGTTCATGGTCAGTTGC	3' *gliZ* for qRT-PCR
oligo 303	GCTACCACTCTGCATCCTCA	5' *abaA* for qRT-PCR
oligo 304	TACGAGCTCCAGCATGATTC	3' *abaA* for qRT-PCR
oligo 305	ACGGCAGGAAGTTGTCTTCT	5' *wetA* for qRT-PCR
oligo 306	CTGTCAGCGACTTGTTGGAT	3' *wetA* for qRT-PCR
oligo 319	GGGATCTGATTCACATGCAG	5' *laeA* for qRT-PCR
oligo 320	GATGGGAATAAATCCTCCGA	3' *laeA* for qRT-PCR
oligo 346	CCATGTGTGTCGAGTCCTTC	5' *ef1* for qRT-PCR normalization
oligo 347	GAACGTACAGCAACAGTCTGG	3' *ef1* for qRT-PCR normalization
oligo 430	TTCCAAATGTGGCAAGTGAT	5' *acyA* for qRT-PCR
oligo 431	GCAAACGTGGAATCAATACG	3' *lacyA* for qRT-PCR
oligo 434	CCACCACCTACAACAACAGC	5' *pkaC1* for qRT-PCR
oligo 435	TGTGAAGACGCATGATGAGA	3' *pkaC1* for qRT-PCR

oligo 697	GCAATGTAAAGCTAACGTGCGTG	5' *AnpyrG* marker
oligo 698	TGCCTTTAAGCTTCGGGTAGAG	3' *AnpyrG* marker
oligo 1208	TCCAAGCTGATGGAGCAGAA	5' *cyp51A* for qRT-PCR
oligo 1209	GTGAATCGCGCAGATAGTCC	3' *cyp51A* for qRT-PCR
oligo 1210	GCGGGTCAACATTCTTCCTC	5' *cyp51B* for qRT-PCR
oligo 1211	GAGGCAAGTCAGATCCGAGA	3' *cyp51B* for qRT-PCR
oligo 1278	CGCGCAGAGAAGACAACCACAT	5' *stuA* for qRT-PCR
oligo 1279	TGCATAGGGCCAATCTTCACAACA	3' *stuA* for qRT-PCR
oligo 1320	AGGACTGGACACAGTGGATG	5' *srbA* for qRT-PCR
oligo 1321	CTAGGCGAAGCATTTGCAGT	3' *srbA* for qRT-PCR
oligo 1468	GCCCTACGATGTAGAAAGGA	5' flanking region of *elp3*
oligo 1469	*CTGATCTACCCCTTGGAACGCAGCA*CTAACTCGGATAAGCACGAG	3' *elp3* with *AnpyrG* tail
oligo 1470	*TTTGTAGGCTTTGGGCTGTTCACAA*CTGCGACGATGGCAGATC	5' *elp3* with *AnpyrG* tail
oligo 1471	AGTAGACGCTCATTGGACCT	3' flanking region of *elp3*
oligo 1472	GCGACTGAACTTACAGGAGC	5' nested of *elp3*
oligo 1473	GCGGCTAGGTGGATCAATG	3' nested of *elp3*
oligo 1474	CCGAGAGCTTGAGGACAGT	5' flanking region of *gcnE*
oligo 1475	*TTTGTAGGCTTTGGGCTGTTCACAA*CTCTGGCTTCTTCAACATCC	5' *gcnE* with *AnpyrG* tail

oligo 1476	*CTGATCTACCCCTTGGAACGCAGCA*CTTCATTTTGAAGCGACGGAG	3' *gcnE* with *AnpyrG* tail
oligo 1477	GCGAATGCTGGGATGTGTG	3' flanking region of *gcnE*
oligo 1478	CCGTGATGGCGTTGTGTAG	5' nested of *gcnE*
oligo 1479	CGAGGGACCAGCTTCTGTAA	3' nested of *gcnE*
oligo 1561	GCTGCAAGTAGGCGTTTACA	5' *medA* for qRT-PCR
oligo 1562	GCACGGCCATCTTCATATCC	3' *medA* for qRT-PCR
oligo 1563	CCCTCTCGACCAGTACATCC	5' *uge3* for qRT-PCR
oligo 1564	CCAATGTTGTGTGACTGCCA	3' *uge3* for qRT-PCR
oligo 1581	GCAAGAAAGGCAACAGCAAC	5' *somA* for qRT-PCR
oligo 1582	GCATATTGAGCGGACCCATC	3' *somA* for qRT-PCR
oligo 1663	GTCAATTCCCAACGGCTCTC	5' *gfaA* for qRT-PCR
oligo 1664	ACGCCATGTTTCAGTTCACC	3' *gfaA* for qRT-PCR
oligo 1691	*TAG TTC TGT TAC CGA GCC GG*C TCG TGC TTA TCC GAG TTA G	5'*elp3* with *hygB* tail for complementation
oligo 1692	*GCT CTG AAC GAT ATG CTC CAA C*CG TCT TGT CTT TGT TTG ATG TC	3'*elp3* with *hygB* tail for complementation
oligo 1693	*TAG TTC TGT TAC CGA GCC GGC* TCC GTC GCT TCA AAA TGA AG	5'*gcnE* with *hygB* tail for complementation
oligo 1694	*GCT CTG AAC GAT ATG CTC CAA C*AT ACC CAA TGG ATG CAT GGG	3'*gcnE* with *hygB* tail for complementation

[a] Tail sequence is in italic.
